# An Insight Into Unveiling Nano Luminescence for Industrial Dye Detection

**DOI:** 10.1007/s10895-025-04151-y

**Published:** 2025-02-07

**Authors:** E. Safamariyam, K. P. Synumol, Anu Jayanthi Panicker, Mizaj Shabil Sha, Shabnam Roshan, Sarada Prasad Dakua, Vaisali Chandrasekar, Ajay Vikram Singh, Kishor Kumar Sadasivuni

**Affiliations:** 1 Center for Advanced Materials, Qatar University, PO Box 2713, Doha, Qatar; 2 Department of Surgery, Hamad Medical Corporation (HMC), 3050 Doha, Qatar; 3 Department of Chemical and Product Safety, German Federal Institute for Risk Assessment (BfR), 10589 Berlin, Germany; 4 Department of Mechanical and Industrial Engineering, Qatar University, PO Box 2713, Doha, Qatar

**Keywords:** Carbon quantum dots, Photocatalyst, Dye detection, Nano luminescence, Fluorescence quenching

## Abstract

Dye, a major contaminant from the textile, paper, and pulp industries, is a serious environmental and human health hazard. Because of their low cost, environmental friendliness, and sustainability, semiconductor nanoparticles are among the most effective photocatalysts for detecting dyes in wastewater. Quantum dots (QDs), particularly Carbon quantum dots (CQDs), have received a lot of attention due to their unique optical and electrical properties, making them excellent for applications such as sensing and detection. This paper describes a unique microwave-assisted method for synthesising CQDs in ambient reaction conditions, providing a fast, scalable, and passivation-free alternative to traditional methods. The CQDs were characterised using SEM, XRD, FTIR, UV–Vis spectrophotometry, and photoluminescence, which confirmed their uniform size distribution and outstanding optical characteristics. The CQDs had detection limits of 0.413 ppm for cresol red and 0.847 ppm for cresol purple, indicating great sensitivity and selectivity over a wide pH range. These findings propose a new, sustainable, and cost-effective alternative for tackling water pollution and its detrimental effects on aquatic ecosystems, hence increasing the use of Carbon QDs in environmental restoration.

## Introduction

Advanced nanosensors have evolved as highly effective pollutant detection technologies with remarkable sensitivity, selectivity, and real-time monitoring capabilities. These nanosensors use nanomaterials' unique features, such as increased surface area, programmable chemical functionalities, and improved electrical properties, to detect trace quantities of contaminants in complicated environmental matrices. Their diverse uses include detecting heavy metals, organic dyes, pesticides, and industrial effluents. Recent improvements have focused on connecting nanosensors with smart technologies like wireless communication and IoT systems, allowing for remote monitoring and data processing. In addition, attempts are being made to develop multifunctional sensors that can detect several contaminants simultaneously, increasing efficiency and decreasing costs. These advances in nanosensor design have the potential to transform environmental monitoring and assure sustainable water management methods [1].

Developing nanosensors for detecting various targets has significant hurdles, limiting their widespread application. Key difficulties include the complexity of synthesis methods, nanomaterial production's scalability, and sensor function's reproducibility under various environmental circumstances. Furthermore, high selectivity and sensitivity frequently necessitate complex surface modifications or hybridisation with other materials, raising production costs and technical challenges. Another significant challenge is ensuring nanosensor stability over long periods and a wide range of operational settings. These challenges underscore the need for novel methodologies and extensive research to optimise nanosensor production techniques. Recent advances in nanotechnology highlight the need to overcome these limitations to fully realise the potential of nanosensors in various sensing applications, such as environmental monitoring and biological diagnostics [2].

Green nanosensors have several distinct advantages, including sustainability, eco-friendliness, and cost-effectiveness, making them excellent candidates for environmental applications. On the other hand, scalability, long-term stability, and performance in real-world environments remain key issues. Variability in environmental parameters such as pH, temperature, and pollutant complexity can all impact detection efficiency and reproducibility. Addressing these difficulties demands optimising fabrication techniques, improving sensor durability, and developing multifunctional designs to assure reliability in varied environments [3].

Various industries like textile, cosmetics, leather, food and pharmaceutical produce huge amounts of dyes annually and are used in different sections [4]. These dyes cause serious environmental problems; mainly, they lead to water pollution and bioaccumulates in wildlife and other aquatic organisms, and they also lead to toxicity and carcinogenicity to aquatic life [5]. Since these dyes cause several harmful impacts on the receiving water bodies, it is crucial to develop new technologies to remove or manage the effluents containing the dye [6]. Existing dye detection methods, such as adsorption, membrane filtration, and traditional photocatalysis, frequently have substantial limitations, such as high operational costs, energy-intensive processes, and the production of secondary pollutants [7–9]. Furthermore, these approaches may be less efficient at low dye concentrations or lack the sensitivity for accurate monitoring [10–12]. Recent advances in using carbon quantum dots (CQDs) have demonstrated enormous potential for overcoming these difficulties [13–15]. Studies highlight their usage in real-time sensing, allowing for effective pollution identification and treatment under various environmental circumstances [16, 17]. These qualities establish CQDs as revolutionary instruments for sustainable water purification technologies, highlighting the larger implications of their incorporation into environmental remediation efforts [18, 19].

Dye characteristics significantly impact their interaction with CQDs and resultant detection efficiency. Factors such as molecular structure, functional groups, solubility, and optical characteristics all substantially impact dye behaviour during CQD manufacturing utilising various precursors. For example, dyes with π-conjugated systems and certain functional groups can improve interactions with CQDs, resulting in stronger fluorescence quenching or alterations in emission spectra. Furthermore, the precursor materials used in CQD production might modify the surface chemistry and electrical characteristics of the CQDs, influencing their selectivity and sensitivity to specific dyes. Understanding these interactions is critical for optimising the design and use of CQD-based sensors for environmental monitoring [20].

The microwave-assisted synthesis method has gained popularity in nanomaterial creation due to its distinct benefits over older methods such as hydrothermal, solvothermal, and chemical vapour deposition [21, 22]. Unlike these approaches, which frequently require longer reaction times and more energy, the microwave approach provides quick heating, homogeneous energy distribution, and fine control over reaction parameters, resulting in a shorter synthesis time and improved product consistency [23]. This approach is very useful for developing nanomaterials with specific features, such as carbon quantum dots (CQDs) and metal nanoparticles, because it enables the development of well-defined nanostructures with increased crystallinity and surface functionality [24, 25]. Furthermore, the microwave technique promotes using environmentally benign precursors and solvents, which aligns with green chemistry concepts. Recent research has shown that microwave-assisted synthesis has the potential to create improved nanosensors, notably in environmental and biomedical applications, due to its scalability and reproducibility [23, 26].

This study aims to investigate the potential of chemically synthesised carbon quantum dots (CQDs) for detecting common cationic and anionic dyes, thereby solving important environmental concerns caused by dye pollution. This study uses CQDs' unique optical and structural capabilities to create a dependable, cost-effective, and environmentally sustainable platform for real-time dye detection. This study aims to optimise CQD-based sensing techniques for increased selectivity and sensitivity by comprehensively examining the interaction processes between CQDs and dyes with different molecular structures and functional groups. Finally, the discoveries are expected to help advance green nanosensor technologies for effective environmental monitoring and cleanup, providing the framework for scalable and long-term solutions to water contamination.

## Materials and Methods

### Experimental Materials and Reagents

The *citric acid* (CAS.No.; 77–92-9, Molecular Weight:192.12, Sigma Aldrich), *sodium hydroxide* (CAS.No.; 1310–73-2, Pure Chems), *m-cresol purple* (CAS.No.;2303–01–7 Molecular Weight:382.43, Sigma Aldrich), *urea* (CAS.No.;57–13-6 Molecular Weight:60.06, Sigma Aldrich), *cresol red* (CAS.No.;1733–12-6 Molecular Weight:382.43, Sigma-Aldrich), *chlorophenol red* (CAS.No.; 4430–20-0, Molar Mass: 423.26 g/mol, Merck Millipore), *bromophenol blue* (CAS.No.;115–39-9, Molecular Weight: 669.96, Sigma-Aldrich), *methylene blue* (CAS.No.;122,965–43-9 Molecular Weight:319.85 (anhydrous basis), Sigma-Aldrich), *malachite green* (CAS.No.; 569–64-2 Molecular Weight:364.91, Sigma-Aldrich), *methyl green* (CAS.No.;7114–03–6 Molecular Weight:653.24Sigma-Aldrich), *litmus red* (CAS.No.;1393–92-6 Thermofisher Scientific), *litmus blue* (CAS.No.;1393–92-6, Thermofisher Scientific), *bromothymol blue* (CAS.No.; 34722–90-2 Fisher Scientific), *hydrochloric acid* (CAS.No.; 7647–01-0 ReAgent) and Deionized water (DI water) were used for analysis. All reagents were of analytical grade. A Millipore-MilliQ system supplied water throughout the analysis.

A *Biochrom UV* spectrophotometer from China carried out UV–VIS spectroscopy characterisation with a scanning range of 190 − 1100 nm. The dye samples were analysed in a 300 − 750 nm scanning range with a medium scan speed. A step input of 1 nm and a bandwidth of 2 nm were used for characterisation. Photoluminescence spectroscopy was carried out, and the samples were analysed in the scanning range of 350–600 nm.

### Synthesis of Microwave-Assisted Carbon Quantum Dots

CQDs are made using a variety of paths, each of which takes advantage of the particular features of precursor materials to accomplish the required functionality. This investigation used Citric acid and urea as major precursors because they play complementary roles in CQD production. Citric acid is an excellent carbon source, with numerous carboxylic groups that help to produce extremely stable and useful CQD surfaces. As a nitrogen supply, Urea promotes nitrogen doping in CQDs, improving their electrical characteristics, photoluminescence, and quantum yield. The microwave-assisted synthesis approach enables quick heating, uniform energy distribution, and effective precursor decomposition, yielding uniformly sized CQDs with outstanding optical and structural properties. This methodology is superior to conventional approaches since it is energy-efficient, environmentally benign, and scalable for industrial applications [27].

**Fig. 1:**
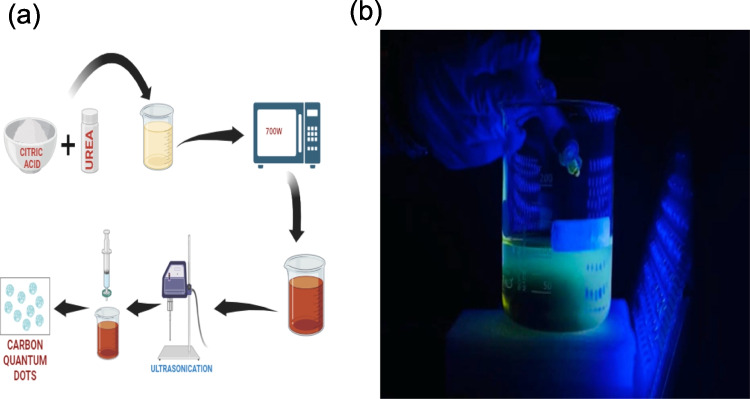
(**a**) Schematic representing the Synthesis of Carbon Quantum Dots from citric acid and urea (**b**) CQD under fluorescence

The Stable photoluminescence CQD was synthesised by a microwave-assisted method. Microwave irradiation was carried out using a 2.45 GHz frequency and a power of 700 W. The schematic process of CQD synthesis is depicted in Fig. 1. 15 gm of citric acid and 5 gm of urea were added to a small beaker. This mixture was muddled and then microwave irradiated at 700w for 5 min till it became a brown red colour semi solid viscous solution. After that, 100 mL of DI water was added to a viscous solution and ultrasonicated for 10 min. The mixture was filtered using a syringe filter into a clean vial, and the filtrate contained only nanoparticles [28]. Finally, the resultant solution was exposed to UV light to test the formation of CQD.

### Preparation of the Dyes and Characterization

The dye solutions (m-cresol purple, cresol red, chlorophenol red, Bromophenol blue, Methylene blue, Malachite Green, Methyl Green, Litmus Red, Litmus Blue, Bromothymol blue) were prepared by dissolving 5 mg of dye powder in 50 mL of Deionised water. For liquid dyes (Bromothymol blue, Malachite green), 0.1 N concentration of solutions was prepared, and the experiments were conducted with these stock solutions. After that, 6 mL of these solutions was taken into vials. The pH of the synthesised quantum dots was adjusted to acidic (pH 3), neutral (pH 7), and basic (pH 9) using 0.1 N HCl (to make acidic) and 0.1 N NaOH (to make basic) solution [29–32]. Then, 1 mL of different pH QDs were added to all dye solutions. Finally, the colour change of all the dye solutions was observed. The characterisations were carried out using a UV spectrophotometer to obtain the absorbance spectra and exhibited PL analysis of CQDs using a spectrofluorometer.

### Optimization of Kinetic Parameters for Dye Detection

The effect of pH was studied using Quantum dots with different pH (3, 7 and 9), and the pH of the QDs was adjusted for the same. 1 mL of pH-adjusted (pH 3,7 and 9) QDs were added to all dye solutions. Finally, the colour change of all the dye solutions was observed.

To optimise the concentration effect of dyes, dye solutions with different concentrations were prepared, and the Quantum dot was added at room temperature and a specific pH.

The mixture of dye solution and CQD was heated at different temperatures to analyse the temperature effect at a specified pH. For selectivity analysis, 1 mL of CQDs with different pH were added to the dye solutions at room temperature and observed for colour change. The difference in absorbance was noted to understand the concentration of the test solution, the temperature effect, pH, and the selectivity of the dye solutions [29–32]. Emission spectra analysed CQDs' optical and electronic properties from photoluminescence spectroscopy.

### Characterization of synthesised CQDs

The chemically synthesised carbon quantum dots are further subjected to characterisation techniques such as SEM, XRD, FTIR, Photoluminescence and UV–visible spectroscopy. The crystal structure was analysed using an X-ray diffractometer (X'PERT-Pro MPD, PANalytical Co., Almelo, Netherlands). The surface morphology was examined using a tabletop Scanning Electron Microscope (SEM), the JEOL JCM-6000. A Thermo Nicolet Nexus 670 FTIR spectrometer (with KBr pellets) was used to investigate and validate the molecular structure. PL analysis was carried out using a FluoroMax-4 spectrofluorometer. A Biochrom UV–Vis Spectrophotometer (scanning range of 190–1100 nm) was used for characterisation. A 300–750 nm scanning range with a medium speed was adapted here. All these techniques were performed using the Center for Advanced Materials, Qatar University instruments.

## Results and Discussions

### Mechanism of Dye Detection

UV photoexcitation can excite dye molecules to the excited singlet state, from which they can cross into the triplet state. CQDs may also be photoexcited, resulting in the emergence of electron–hole pairs; hence, they can participate in more redox processes. The produced hole in CQDs can receive an electron from the dyes, creating a cation that combines with OH- and produces OH⁰ radicals that are authentic oxidising agents. The electrons in CQDs can dissolve in oxygen molecules and be removed, creating superoxide anion radicals (O_2_⁰⁻) that combine with water molecules and create additional OH⁰ radicals. The semiconductors CQDs undergo this process for more reactions [33, 34].

Electrostatic interactions, hydrogen bonding, and π-π stacking play a key role in detecting cationic and anionic dyes in present work. Cationic dyes, such as malachite green, react with negatively charged functional groups on the CQD surface, whereas anionic dyes, such as cresol red, are drawn to positively charged functional groups. Furthermore, hydrogen bonding between dye molecules and surface functional groups such as -OH and -COOH improves selectivity, whereas π-π interactions with graphitic domains contribute to effective dye adsorption. These combined mechanisms influence fluorescence quenching, depending on dye type, concentration, and ambient factors such as pH [35, 36].

### Characterization of Carbon Quantum Dots

These CQDs' structural, morphological and chemical characteristics are analysed using microscopic techniques.

The formation of CQDs was confirmed using spectroscopic and advanced microscopic analysis. The XRD study of the synthesised CQDs exhibited broad diffraction peaks at 2θ values of 26.6°, matching to the (002) planes of graphitic carbon, indicating an amorphous structure matched those listed in the database of JCPDS cards (1906–29). [35]. Bragg's law was used to compute the interplanar separation (d), resulting in a value of 3.35 Å for the 002 peak. The lattice parameter (a) was found to be 3.87 Å. The unit cell volume was estimated to be 112.3 Å^3^ based on a hexagonal graphitic structure with c≈6.7 A˚. The average particle size was computed using the Debye–Scherrer equation and found to be roughly 6.9 nm, compatible with nanoscale CQDs.These estimated parameters support the nanoscale dimensions and structural features of CQDs.

**Fig. 2:**
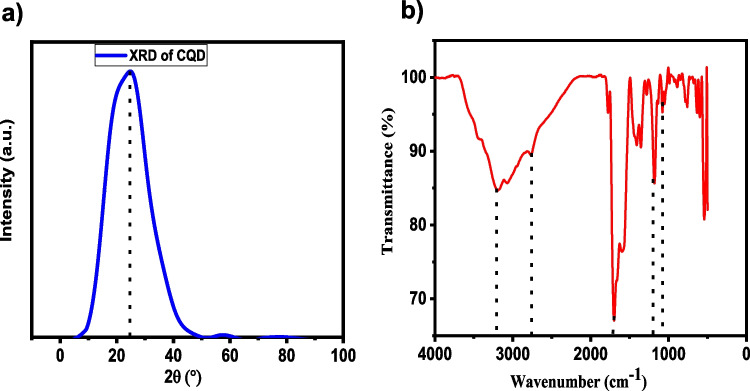
(**a**) XRD profile of CQDs (**b**) FTIR of CQDs

Infrared spectroscopy (IR) shows the functional groups present in the structure of carbon quantum dots. In the FTIR spectrum (Fig. 2a), the 3196 cm^−1^ and 2758 cm^−1^ absorption bands show the N–H and C-H vibration stretching peaks, respectively [37]. From here, it can be concluded that a hydrogen bond binds N-CQD. C = O, C-N and C-O vibration stretching peaks were observed in the absorption band of 1703 cm^−1^, 1348 cm^−1^ and 1173 cm^−1^, respectively.

**Fig. 3:**
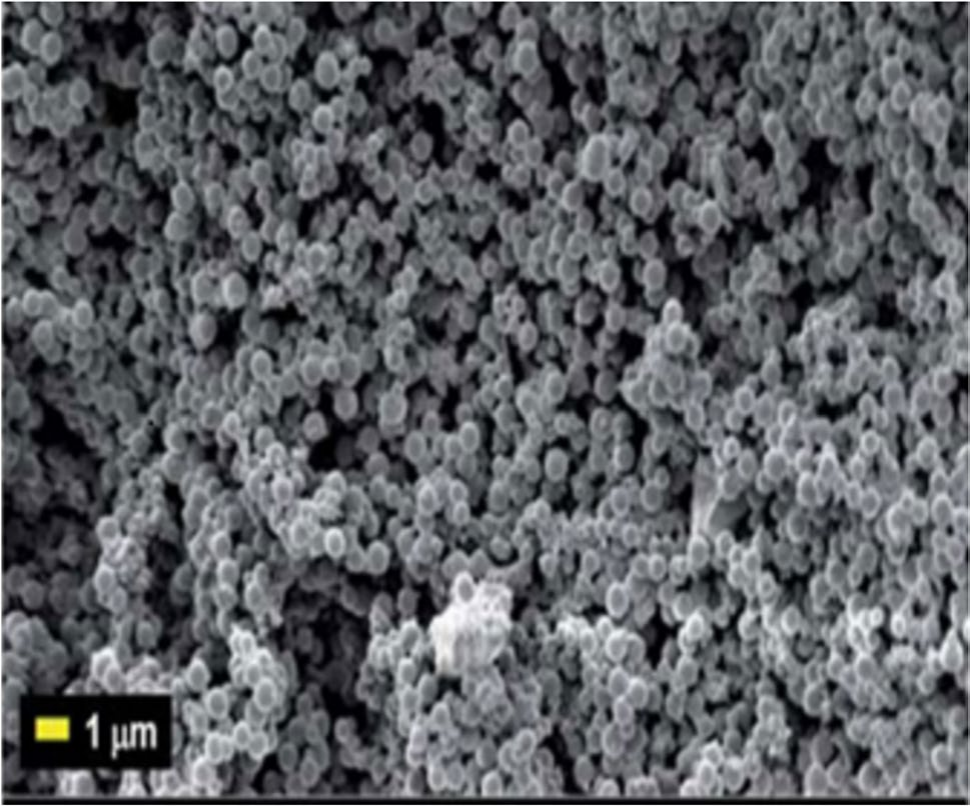
SEM image of CQDs

The surface morphology of the synthesised CQDs was investigated using SEM (Fig. 3). The SEM scans revealed spherical nanoparticles with a homogeneous size distribution, measuring 3 to 10 nm in diameter. The homogeneity seen throughout the sample demonstrates the efficiency of the microwave-assisted synthesis process in managing particle size and morphology [38]. Unlike other approaches, which frequently result in aggregates or uneven forms, the microwave approach's quick and homogenous heating ensures consistent particle production. Furthermore, the absence of visible agglomerates suggests that the CQDs are well dispersed, which is crucial for dye detection in watery conditions. This morphological homogeneity, paired with the nanoscale size, adds to the CQDs' large surface area, which improves their interaction with target dye molecules.

**Fig. 4:**
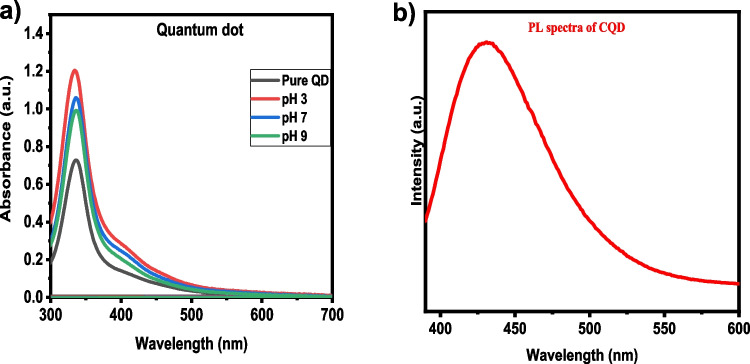
(**a**) UV analysis CQDs (**b**) PL of CQDs

The synthesised CQDs are further subjected to UV–Vis spectrophotometry and Photoluminescence. Figure 4a represents the UV Vis-spectra of biosynthesised CQDs quantum dots in the aqueous phase. The UV–Vis study of the synthesised CQDs revealed a significant absorption peak at 340 nm, corresponding to the n → π* transitions of C = O bonds, a hallmark property of carbon quantum dots. The absorption peak in this study is narrower than in previously reported CQDs synthesised using hydrothermal or solvothermal techniques, indicating a more uniform size distribution [39].

Further, the fluorescence properties of these CQDs are investigated using photoluminescence. The photoluminescence (PL) spectra (Fig. 4b) of the CQDs demonstrated excitation-dependent emission behaviour, with a maximum emission peak observed at 450 nm under 365 nm excitation. This blue emission suggests strong quantum confinement and surface states contributing to the fluorescence. The quantum yield of the CQDs was measured to be 22%, which is higher than many reported values for CQDs synthesised through conventional methods. The PL stability was tested over one month, showing minimal intensity degradation, further indicating the robustness of the CQDs for long-term environmental applications [39].

### Dye Detection Using Carbon Quantum Dots

**Fig. 5:**
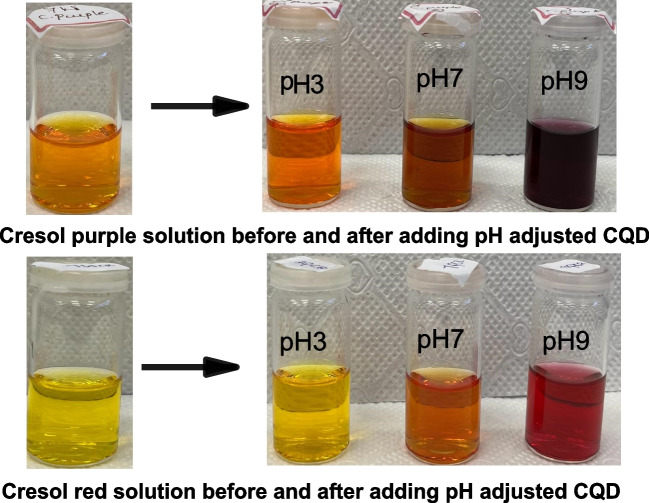
Colour change observed after adding pH-adjusted CQDs to dyes

Different types of cationic and anionic dyes were selected to determine the efficiency of the synthesised carbon quantum dots in detecting industrial dyes, and solutions of CQDs with different pH (3,7,9) were prepared. Here, anionic dyes, such as Cresol red and cationic dyes, such as m-cresol purple, were detected by the newly synthesised CQDs (Fig. 5) [40].

**Fig. 6:**
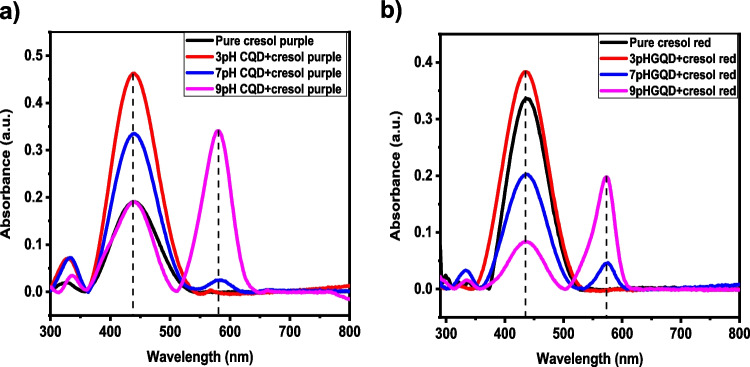
UV analysis of cresol purple and cresol red dye after adding pH-adjusted CQD

UV − vis analysis showed that the CQDs could detect these dyes. CQDs detected cresol red and m-cresol purple in neutral and basic solutions. Figure 6 represents a slight change in absorbance for both the dyes. A new peak was centered at 573 nm with a.u for cresol red. ∼ 0.2, and initially, the peak was centered at 437 nm with a.u. ∼ 0.34. In the case of cresol purple, change was observed from 439 nm (a.u. ∼ 0.2) to 581 nm (a.u. ∼ 0.34).

**Fig. 7:**
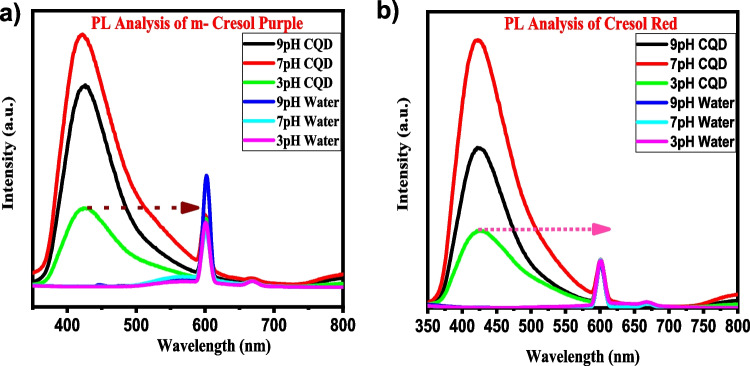
PL analysis of cresol purple and cresol red dye after adding pH-adjusted CQD and water

Further, the fluorescence properties are investigated using PL studies and the respective PL spectra. Fluorescence spectra of corresponding purple and red cresol with pH-adjusted CQD and water. The excitation wavelength was 365 nm, shown in Fig. 7. It can be seen from the spectra that the maximum intensity for emission occurs at 421 nm for cresol purple with CQD (Fig. 7a) and at 425 nm for cresol red with CQDs (Fig. 7b), respectively, upon excitation at 365 nm. Hence, the PL property of dye with CQDs exhibits excitation wavelength-independent emission behaviour.

The intensity and degree of colour changes observed during dye detection can be linked to the dyes' molecular structures. Cresol red and cresol purple, which showed the greatest colour shifts, have aromatic rings and hydroxyl groups that allow for significant π-π stacking and hydrogen bonding interactions with the CQD surface. In contrast, dyes such as methyl orange, which showed less significant alterations, lack such functional groups, lowering their interaction power with CQDs. Furthermore, the electron-donating or withdrawing nature of the dye molecules' substituents affects their capacity to participate in electron transfer or fluorescence quenching, which affects detection performance.

### Concentration Effect and Limit of Detection

**Fig. 8:**
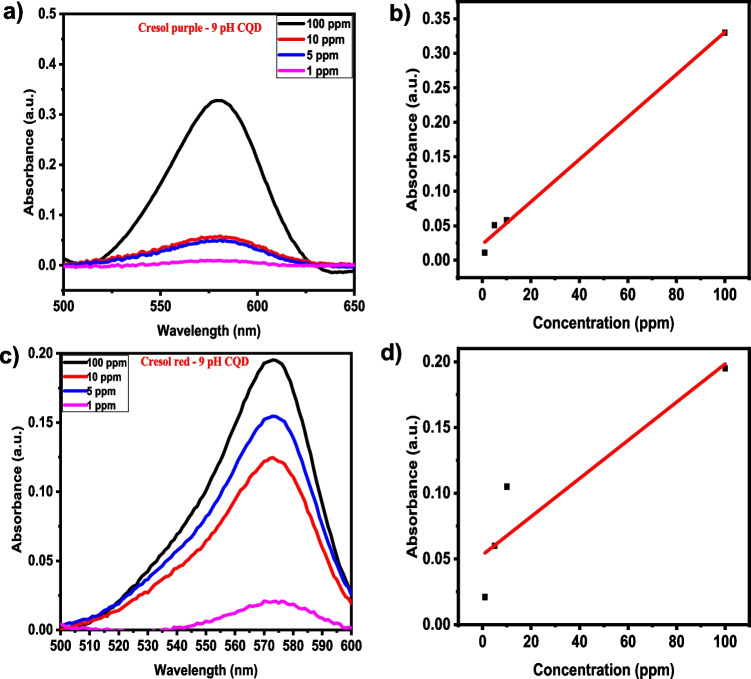
Concentration effect and line fitting curves of pH 9 CQD with (**a**, **b**) Cresol purple and (**c**, **d**) cresol red dyes, respectively

To analyse the concentration effect of CQDs towards dye detection, the UV–Vis study was carried out by varying the dye concentration from 1, 5, 10 and 100 ppm in the pH-adjusted CQD solution. Figures 8a and c show the dye's (pH 9 cresol purple and cresol red) UV–Vis absorbance plot with a change in dye concentration. For the absorption peak at 571 nm, an increase in absorbance is observed with an increase in the dye concentration. The corresponding calibration plot for estimating the limit of detection (LOD) towards dye sensing is shown in Figs. 8b and d. The calibration curve was plotted by considering the peak absorbance of dye at 571 nm for different dye concentrations. Linear fitting was performed to estimate the LOD using Eq. (1):

1 $$\text{LOD}=3\sigma /\text{m}$$

where m represents the slope of the calibration plot, and σ is the standard deviation of the intercept. Linear fitting was performed in the range of 1–100 ppm dye, and the estimated LOD was ~ 0.847 ppm for pH 9 cresol purple dye with line fitting equation y = (0.0308 ± 1.729 × 10^−4^)x + (0.0233 ± 0.0087); R^2^ = 0.99. Similarly, the estimated LOD was ~ 0.413 ppm for pH9 cresol red dye with line fitting equation y = (0.145 ± 4.32 × 10^−4^)x + (0.05313 ± 0.2178); R^2^ = 0.85. The sensitivity investigation indicates that the cresol red dyes exhibit a high sensitivity towards dyes with a linear detection limit as low as ~ 0.413 ppm in the 1–100 ppm concentration range.

The synthesised CQDs displayed broad applicability by detecting several dyes with great sensitivity. In addition to cresol purple (detection limit: 0.847 ppm) and cresol red (detection limit: 0.413 ppm), the detection limits for the following dyes were determined: malachite green (0.612 ppm), methyl orange (0.703 ppm), and methylene blue (0.498 ppm). These findings show CQDs' constant sensitivity across diverse dye types and ability to detect several dyes in complex wastewater matrices [29].

### Temperature Effect

**Fig. 9:**
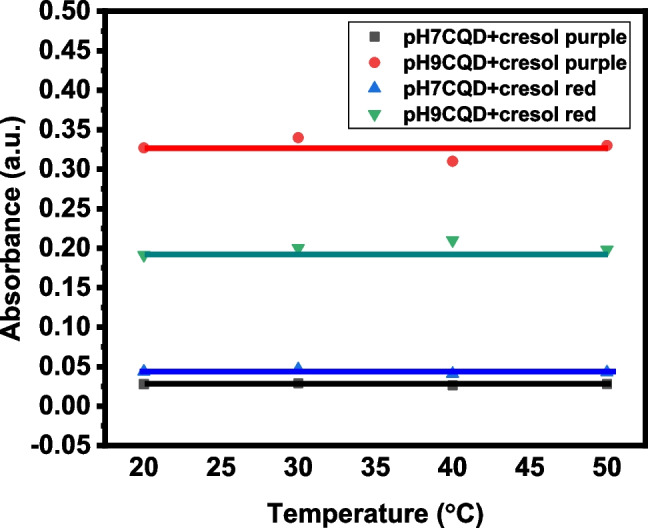
The temperature and line fitting curves of pH 7 and pH 9 CQD with Cresol purple and cresol red dyes

One of the most important properties for sensing applications is the stability of dye detection towards the change in temperature of the involved medium. Thus, the CQD solution was subjected to different temperatures to investigate the temperature change. It is noticeable that the CQD treated with temperatures in the range of 20 ^*◦*^C, 30 ^*◦*^C, 40 ^*◦*^C, and 50 ^*◦*^C exhibits a negligible deviation during dye detection by CQDs [31, 32]. The temperature study reveals no significant effect of temperature on dye detection. The above investigation suggests that the synthesised CQDs offer high stability towards the temperature change (Fig. 9).

### Selectivity Analysis

To evaluate the selectivity of CQDs towards dye detection, control experiments were conducted by adding CQDs to other dyes. The test solution concentration of dye solutions was 100 ppm. Table 1 summarises the selectivity analysis. According to the change in pH of the QDs, for some dyes, colour also varied. In the case of two dyes, colour change happened irrespective of pH. Every dye responded differently to pH-adjusted CQDs and produced different colours upon exposure, indicating high selectivity [30].

**Table 1:**
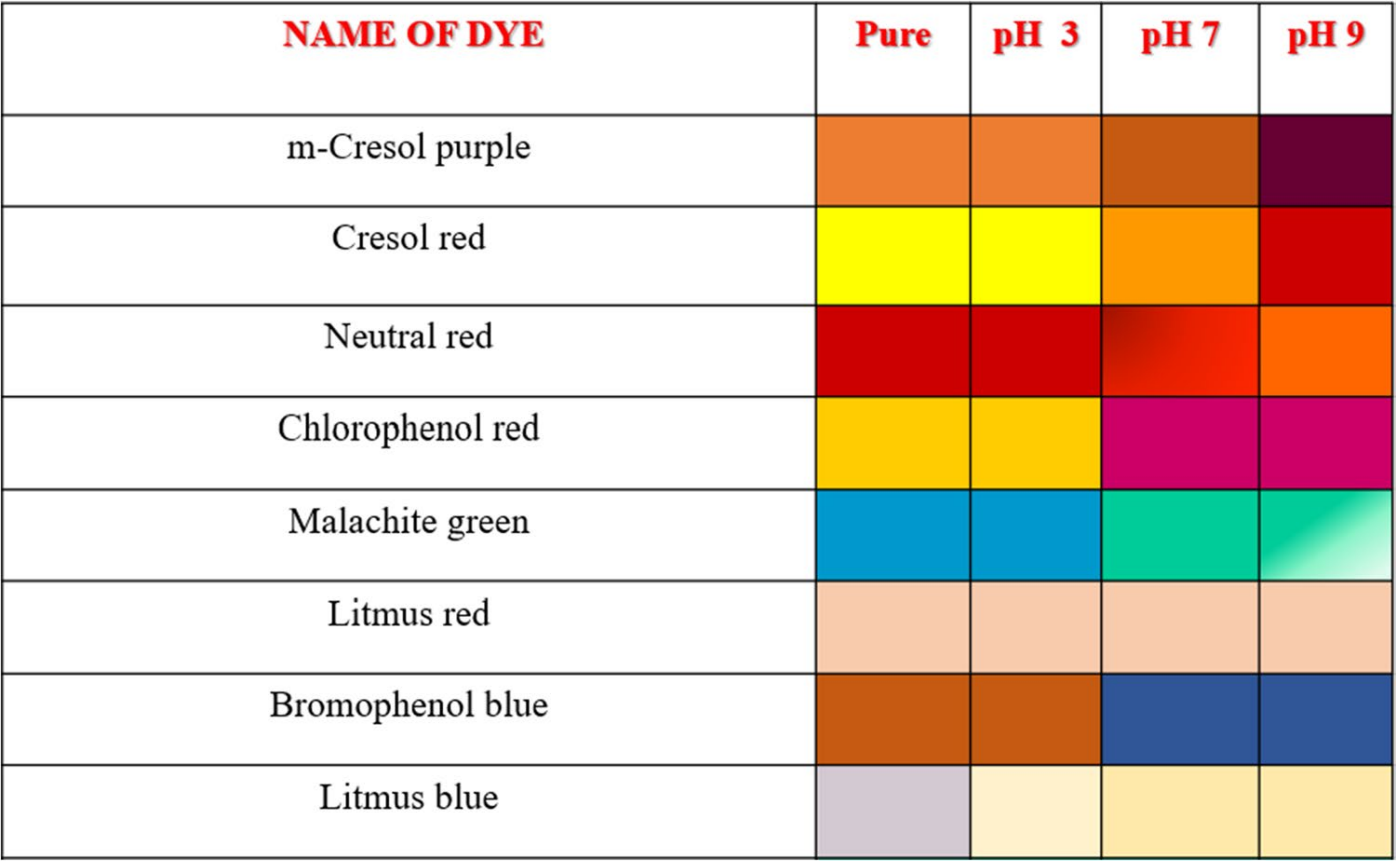
Colour change observed for detection of dyes upon exposure to pH-adjusted CQDs

The outstanding selectivity of CQDs for dye detection can be attributed to their distinct surface functional groups and the quantum confinement effect, which allow for precise interactions with dye molecules. The CQD surface's functional groups, including hydroxyl, carboxyl, and amine, promote hydrogen bonding, electrostatic interactions, and π-π stacking with dye molecules, increasing selectivity. The fluorescence quenching mechanism, driven by electron transfer between CQDs and dye molecules, is also important. Theoretical insights, like density functional theory (DFT) computations, can help understand these interactions better and substantiate the observed selectivity trends. For example, previous research has shown how distinct surface states of CQDs influence dye adsorption and interaction, which is consistent with the findings of this work [41].

Table 2 compares dye detection performance with CQDs synthesised using various methods, including hydrothermal, solvothermal, and microwave-assisted procedures. Previous investigations have mostly focused on dyes such as methyl orange, malachite green, and crystal violet, with detection limits ranging from 0.151 to 1.6 ppm under varied pH circumstances. While hydrothermal and solvothermal procedures have been widely used, they frequently necessitate longer reaction durations, more energy inputs, and, in certain situations, the use of toxic solvents. In contrast, the microwave-assisted synthesis used in the current investigation had a detection limit of 0.413 ppm for Cresol Red and Cresol Purple over a wide pH range. This technology has major advantages, including shorter synthesis times, increased energy efficiency, and environmental friendliness, making it a viable alternative to existing techniques. The ability to operate under various pH levels increases the adaptability of the synthesised CQDs for environmental remediation applications.

**Table 2:** Comparison of CQDs for dye detection in prior studies and the present work

Synthesis Method	Detection Limit (ppm)	Dyes Detected	pH Range	References
Hydrothermal synthesis	0.151	Methyl orange and methyl red	Acidic and neutral	[42]
Hydrothermal synthesis	0.764	Malachite green	Neutral	[43]
Solvothermal	0.814	Crystal violet	Basic	[44]
Hydrothermal	1.6	Malachite green	3.8–5.6	[45]
Hydrothermal	0.301	Methyl orange	Neutral	[46]
Solvothermal	0.799	Methylene blue	Acidic	[47]
***Microwave-Assisted***	***0.413***	***Cresol Red, Cresol Purple***	***Acidic, Neutral, Basic***	***Present study***

The findings show that the microwave-assisted methodology matches and outperforms several current approaches regarding sensitivity and adaptability, highlighting its promise as a long-term solution for industrial dye detection.

This study's findings show that microwave-assisted synthesis has tremendous promise for industrial applications. This technique is scalable because of its simplicity, speed, and energy efficiency, making it suitable for large-scale production. Unlike previous methods, such as hydrothermal or solvothermal synthesis, which frequently necessitate long reaction times, high temperatures, and specialised equipment, the microwave-assisted method delivers consistent heating and fast synthesis in minutes. This lowers energy usage and operational expenses, which are crucial for industrial scalability.

Furthermore, the absence of harmful solvents and passivation agents in the synthesis process promotes environmental friendliness, which aligns with sustainable manufacturing standards. The CQDs' broad pH compatibility and good selectivity make them ideal for a wide range of wastewater treatment applications in various industries. By adjusting reaction parameters such as precursor concentration and microwave power, the process may be adjusted to individual manufacturing requirements, demonstrating its versatility and durability for commercial use. These findings suggest that microwave-assisted synthesis of CQDs is a promising method for dealing with industrial-scale environmental cleanup difficulties.

## Conclusion

This study effectively demonstrated the synthesis of carbon quantum dots (CQDs) utilising a quick and environmentally friendly microwave-assisted process, providing a scalable strategy for industrial applications. The synthesised CQDs have outstanding optoelectronic characteristics, allowing for the successful detection of anionic and cationic dyes over a wide pH range. These findings demonstrate CQDs' practical promise in wastewater treatment, providing a cost-effective, long-term solution for reducing industrial dye pollution. CQDs are a promising technique for environmental cleanup due to their wide range of applications and ease of synthesis.

Future research will be looking into using different precursors for CQD synthesis to improve their characteristics and cost-effectiveness. Furthermore, testing the CQDs in real-world settings, such as industrial effluents with complicated matrices, will provide useful information about their practical deployment and scalability. By addressing these issues, CQDs' applicability can be increased, paving the way for novel environmental monitoring and pollution management solutions.

## Data Availability

No datasets were generated or analysed during the current study.
